# Hachish in the Old Testament

**Published:** 1903-10-17

**Authors:** 


### Hachish in the Old Testament.

Dr. C. Creighton contributes an extremely interesting article to a recent number of "Janus" on the indications of the hachish vice in the Old Testament. The use of hachish, the disreputable intoxicant drug of the East, like that of opium, the respectable narcotic, is of unknown antiquity. In the Old Testament there are some half-dozen passages where a cryptic reference to hachish may be discovered and in two at least of these passages the allusion is made easy by the use of a significant word in the Hebrew text. This word is designedly mistranslated in the Vulgate and in the modern versions it is confessedly unintelligible. The first passage is Canticles v. 1. "I am come into my garden, my sister, my spouse: I have gathered my myrrh with my spice: *I have eaten my honey-comb with my honey*: I have drunk my wine with my milk." In the Hebrew text the phrase in italics reads "I have eaten my wood (yagar) with my honey (Debash)." St. Jerome in the Vulgate translated the Hebrew word meaning "wood" by favum or honey-comb; whilst the LXX adopted a similar license by translating the passage "I have eaten my bread with my honey." The term yagar, however, is employed in many other places in the Old Testament and always in the sense of wood, forest, planted field, herbage, or the like, and when real honey-comb is meant the Hebrew word is either tzooph or nohpheth. The meaning, therefore, of the passage in Canticles is clear enough in its aphrodisiac context: "I have eaten my hemp with honey," and this is still the elegant way of taking hachish in the East. The meaning of yagar (wood) in association with debash (honey) is made clear by I. Samuel, xiv, 27, when Jonathan dipped the point of his staff into "honey-wood," and, merely tasting the honey, his eyes were enlightened. The one passage refers to the aphrodisiac effect of hachish, the other to its bellicose or furious effect. The correct exegesis of I. Samuel xiv, 25–45 is of great importance not only for understanding Jonathan's breach of a certain taboo but also for the whole career of his father Saul, ending in his deposition from the kingship through the firm action of Samuel, and the pitiable collapse of his courage on the eve of the battle of Gilboa. The theory is that both Saul and Jonathan were hachish eaters: it was a secret vice of the palace while it was strictly forbidden to the people: Saul had learned it of the Amalekites: it was this vice and not his disobedience in saving captives and cattle alive, which was his real transgression and the true ground of his deposition at the instance of the far-seeing prophet. No true statesman would have taken action on account of a merely technical sin of disobedience: the disobedience was real and vital, but the substance of it had to be veiled behind a convenient fiction. One great object of Jewish particularism was to save Israel from the vices that destroyed the nations around; and Samuel appears in this respect as the first and greatest of the prophets, the prototype *censor morum*.

The incident related in I. Sam. xiv. arose during a raid upon the Philistines in which Jonathan distinguished himself by the number of the enemy he slew, but at the same time broke a certain law or taboo for which he was afterwards put upon his trial and condemned to death. We are told that previous to the slaughter of the Philistines "all (they of) the land came to a wood and there was honey upon the ground. And when the people were come into the wood, behold the honey dropped; but no man put his hand to his mouth, for the people feared the oath. But Jonathan heard not when his father charged the people with the oath; wherefore he put forth the end of the rod that was in his hand and dipped it in an honey-comb (yagarah hadebash) and put his hand to his mouth, and his eyes were enlightened." The Vulgate again translates the Hebrew words signifying "honey-wood" by the misleading term honey-comb though the Syriac version gives a more intelligible account in the words *Et sylvas ingressi essent, essetque mel in sylva super faciem agri, flueretque mel*: expressing not inaptly a field of hemp with the resinous exudation upon the flower stalks which would flow or run by heat. Indeed, Churrus is collected in Central India during the hot season by men clad in leather running violently through the hemp fields. The soft resin adheres to the leather, and is subsequently scraped off and kneaded into balls. The whole incident in the Old Testament is obviously dramatised or made picturesque: the growing field of hemp, the men passing through it, Jonathan dipping the end of a rod or staff into the resin upon a stalk as he passed by. The real meaning is that Jonathan was a hachish eater, and the effect upon Jonathan on this occasion was that he ran amok amongst the Philistines.

The evidence that Saul was a hachish eater is not so direct as in the case of Jonathan. There is not a hint of it until after the incident of the forbidden honey, but in the inquiry which followed it is significant that in the trial by lot Saul and Jonathan are ranged together on one side and the people upon the other. The violent antipathy of Samuel towards Agag leads to the presumption that this king had corrupted Saul, for the Amalekites were the tribe of Arabs who were engaged in the carrying trade between the Arabian Gulf and Lower Egypt or the Mediterranean. They had in their hands the trade in gold and spices and drugs: probably the same Arabs among whom the name of Hachashin was found in the mediæval period, and from whom the Latinised name of assassini was brought to Europe by the returning Crusaders. The appearance of David as a harper to soothe Saul when he was possessed of an evil spirit also points to the use of hachish, for Dr. Moreau ("Du Hachish et de l'Aliénation Mentale") has shown that the only kind of mental aleniation influenced by music is that due to intoxication by hachish. The sudden throwing of the javelin at David whilst he played before the King is a very graphic representation of the ungovernable fits of temper which hachish produces, whilst the most significant and the most pathetic symptom of all is Saul's failure of courage on the night before the battle of Gilboa.

